# The state of health and health services in Sudan as a result of the war

**DOI:** 10.4102/phcfm.v15i1.4260

**Published:** 2023-09-05

**Authors:** Abdelhaleem E. Taha

**Affiliations:** 1 Sudan Medical Specialisation Board, Khartoum, Sudan; 2 Public Health Institute, Khartoum, Sudan

Fighting erupted in Khartoum and other parts of Sudan on 15 April 2023. This has impacted the fragile health system that was already suffering economic and managerial difficulties, in addition to prolonged political instability and sanctions. The added burden on the health services is described in the following eight areas:

*Access to public health services:* Access to care is a huge challenge, as many roads are dangerous because of the fighting and many hospitals have been taken over by militia as barracks for their soldiers. Health service infrastructure has also been damaged or destroyed by missiles and shelling.

*Access to private health services:* These same conditions are impacting the private hospitals in Khartoum and 90% of these hospitals are now closed. Health insurance companies have stopped operating because of a breakdown in information technology and the ability to claim or pay for services.

*Inadequate quality of care:* Healthcare workers are also vulnerable to attack or assault and their safety to access healthcare facilities is not guaranteed. Their movements are severely restricted. Many of the senior and more skilled health workers have been displaced to neighbouring districts, leaving only junior staff or even volunteers. The supply chain is also disrupted for medications, intravenous fluids, laboratory reagents, x-ray films and other supplies such as gauze.

*Public health surveillance:* Under these circumstances, with no communications or transport, it is impossible to monitor infectious disease outbreaks or even epidemics. Doctors Without Borders reported a measles outbreak in the White Nile State of Central Sudan among a displaced population, with the deaths of 13 children. The control of vector-borne infectious diseases is also prevented in Khartoum, Western Darfour and most districts of Northern and Central Darfour because of instability and a lack of funding.

*Mental healthcare:* Mental healthcare programmes are also discontinued. Mental health workers provide their services to the displaced people in Gezira and some other states who suffer anxiety and post-traumatic stress disorders as a result of fighting in Khartoum and Darfour.

*The health workforce:* The occupational health programme is almost inactive because of the war and the healthcare workers have not received their salaries since April 2023. Most government ministries work with 50% of their human resources and all government employees have not been paid since April 2023.

*Water and sanitation:* A lot of districts lack a supply of drinking water for many weeks (e.g. Khartoum-North – Alshafa – Arkaweet) either because of a lack of electricity or the water station is itself occupied by the militia.

*Food and international aid:* The United Nations World Food Programme (WFP) temporarily suspended its operations in Sudan after three staff members died and two others were injured during the conflict.

In Sudan, health status and the health system were already fragile, with health indicators being consistently low and enormous disparities existing between urban and rural areas; since the war erupted on 15 April 2023 the situation has become disastrous and even catastrophic.

